# Spatial facilitation by a high-performance dragonfly target-detecting neuron

**DOI:** 10.1098/rsbl.2010.1152

**Published:** 2011-01-26

**Authors:** Karin Nordström, Douglas M. Bolzon, David C. O'Carroll

**Affiliations:** 1 Department of Neuroscience, Uppsala University, PO Box 593, 751 24 Uppsala, Sweden; 2 Discipline of Physiology, School of Medical Sciences, The University of Adelaide, Adelaide, South Australia 5005, Australia

**Keywords:** target detection, motion vision, hypercomplex, response onset, spatial facilitation, response decay

## Abstract

Many animals visualize and track small moving targets at long distances—be they prey, approaching predators or conspecifics. Insects are an excellent model system for investigating the neural mechanisms that have evolved for this challenging task. Specialized small target motion detector (STMD) neurons in the optic lobes of the insect brain respond strongly even when the target size is below the resolution limit of the eye. Many STMDs also respond robustly to small targets against complex stationary or moving backgrounds. We hypothesized that this requires a complex mechanism to avoid breakthrough responses by background features, and yet to adequately amplify the weak signal of tiny targets. We compared responses of dragonfly STMD neurons to small targets that begin moving within the receptive field with responses to targets that approach the same location along longer trajectories. We find that responses along longer trajectories are strongly facilitated by a mechanism that builds up slowly over several hundred milliseconds. This allows the neurons to give sustained responses to continuous target motion, thus providing a possible explanation for their extraordinary sensitivity.

## 1. Introduction

We know from behaviour (e.g. [1–3]) that flying insects have sophisticated mechanisms for rapid detection of targets such as prey, predators and conspecifics, which they track and pursue aerobatically against textured backgrounds. The complex task of tracking small moving targets amidst visual clutter is assisted by specializations of the eye and brain in many animals. These include the fovea of mammals, raptors and jumping spiders, and the acute zone of insect compound eyes (e.g. [4,5]). Subserved by these optical mechanisms, higher order visual neurons, such as cortical hypercomplex (end-stopped) cells, and insect small target motion detectors (STMDs), respond selectively to small moving targets, with little response to larger objects or to wide-field motion [6–8].

The hoverfly *Eristalis* has an optical resolution limit of approximately 1° [9], meaning that each ommatidium (facet) of the compound eye views a patch of space 1° across. Predatory dragonflies have among the highest spatial resolution reported for insects, about 0.25–0.5° in the acute zone [10,11], but this is still far below the fine resolution of the vertebrate single lens eye. Despite these optical limitations, hoverfly STMDs still respond strongly to very small dark targets—just 0.18° square [6]. Targets this small are well below the nominal resolution limit of the compound eye and would thus be blurred by the optics to a very low contrast image (effective contrast below 2%). Similarly, dragonfly STMDs also display high gain to low contrast targets [12]. Furthermore, despite the optical limitations, insect STMDs display similar selectivity to cortical hypercomplex neurons of much larger mammals [13,14]. The underlying neural pathway must thus employ enormous amplification to boost the tiny signals that such small targets generate.

How do insect STMDs achieve massive signal amplification, yet are able to respond so robustly only to small targets, even in complex clutter [6]? Lateral inhibition at several levels of visual processing probably plays a role [15,16], by inhibiting responses to features that do not fit the unique spatial profile of an optimal target. A distinguishing trait of natural target stimuli—be they conspecifics or prey—is that they are likely to fly along continuous paths. This provides the potential for neural mechanisms that enhance target detection by integrating spatially adjacent local motion detector receptive fields. Here, we test this hypothesis using intracellular recordings from the dragonfly centrifugal STMD1 (CSTMD1). We compared the time course of responses with targets commencing within the receptive field, with responses to targets approaching the same location along longer trajectories, and reveal a slow facilitation mechanism to motion onset.

## 2. Material and methods

We recorded intracellularly from CSTMD1 in wild-caught dragonflies (*Hemicordulia tau)* while they were viewing VisionEgg [17] generated small 0.9° targets moving at 55° s^−1^ on a 200 Hz CRT monitor (complete methods in the electronic supplementary material). The targets appeared and instantly started their trajectories within the receptive field. After a position-aligned normalization, we fitted the response onset with a logistic curve:
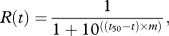
where *t* describes the time, *t*_50_ the time for 50 per cent maximum response and *m* the slope. We fitted the response decay with a one-phase exponential decay:
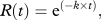
where *k* is the rate constant and *t* the time.

## 3. Results

### (a) Response time course

**Figure 1.**
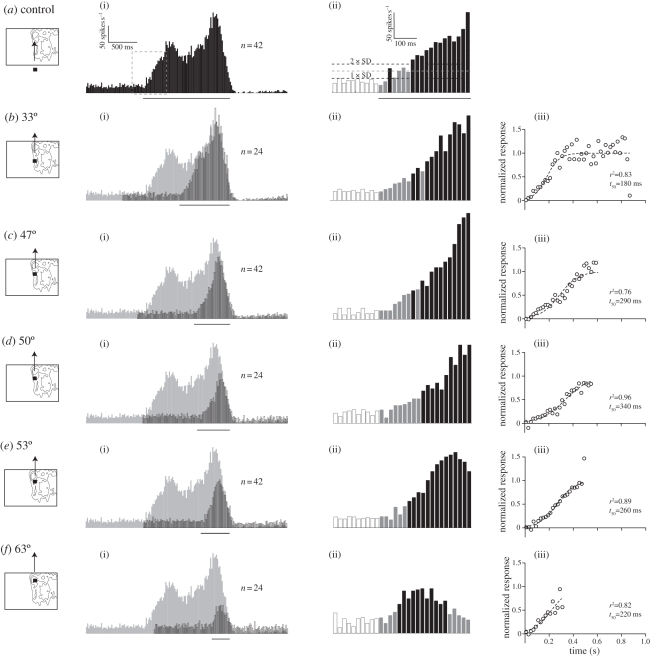
Response time course. (*a*(i)) Spike histogram (*N* = 1, 20 ms bins) showing the response to a 0.9° square black target drifting upwards at 55° s^−1^ through the CSTMD1 receptive field, with the bar underneath indicating peri-stimulus duration. (ii) The magnification surrounding stimulus onset (boxed) uses white bars for pre-stimulus, grey bars for peri-stimulus and black bars for peri-stimulus duration where the spike frequency lies significantly above the spontaneous rate (two-way ANOVA, *p* < 0.05). (*b*(i)) The response (outlined) to a target starting 33° above the display base (pictogram, but note that target is not to scale), with its position-aligned control (from *a*) in grey. (ii) Response surrounding stimulus onset magnified. (iii) Normalized response, fitted with a logistic function (half-time = *t*_50_). (*c*) Response to a target starting 47° above the base. (*d*) Target starting 50° above the base. (*e*) Target starting 53° above the base. (*f*) Target starting 63° above the base.

When a target drifts upwards across the receptive field at 10° azimuth, CSTMD1 responses build up slowly over 400–500 ms to a peak of 150 spikes s^−1^ (figure 1*a*(i)). The second peak corresponds to a hotspot in the receptive field (figure 1*a*(i); electronic supplementary material, figure S1C) associated with the dorsal acute zone [10]. Following the cessation of motion, the neuron shows pronounced post-excitatory inhibition, lasting several seconds.

Following stimulus onset (dashed box, figure 1*a*(i)) we see similar activity to pre-stimulus rates (white bars, figure 1*a*(ii)) for the first 40 ms (grey bars). Consistent with earlier modelling of CSTMD1, suggesting neural delay filters with a short time-constant [12], response rates then increase from 40 ms, and remain significantly above spontaneous rate after 140 ms (black bars, figure 1*a*(ii); two-way ANOVA, *p* < 0.05).

After shifting the display vertically to three elevations (electronic supplementary material, figure S1) the resulting receptive field maps partly reflect the neuron's underlying spatial structure (e.g. the hotspot is located at 60° elevation in all three cases), but the firing rate at the bottom of the display is always low, suggesting a slow response build-up. If we fix the monitor elevation (as for electronic supplementary material, figure S1C), and start the target trajectory within the receptive field, 33° above the display base, the response (outlined, figure 1*b*(i)) rises for *ca* 300 ms before closely matching the spike frequency of its position-aligned control (grey, figure 1*b*(i)). The response onset is very similar to the one for targets traversing the whole screen: initial responses are close to spontaneous rates, and then rise near-linearly for *ca* 400 ms (figure 1*b*(ii)). This slow response onset is not localized to a particular region of the visual field: if we use targets that start closer to the hotspot (figure 1*c*–*f*(i)(ii)), all responses build up slowly for 300–500 ms.

When the target starts nearer the hotspot, the slope of the response build-up is steeper, as the control sensitivity (the underlying receptive field) is higher (e.g. figure 1*c*(ii)). Additionally, in comparisons with shorter target trajectories, the *temporal* effects differ, since accumulation of post-excitatory inhibition is activity dependent (compare figure 1*a*(i) with *f*(i)). This inhibition is apparent following all trajectories, except the shortest (310 ms, figure 1*f*(i)). During longer target trajectories, the neuron is thus subjected to two counteracting forces: response facilitation, which takes hundreds of milliseconds to build to full effect, and an activity-dependent inhibitory depression.

We can normalize for the receptive field's underlying *spatial* structure by dividing the response to targets commencing within the receptive field (outlined, figure 1*b*–*f*(i)) with their position aligned controls (grey, figure 1*b*–*f*(i)). The control represents the response to targets that start their trajectory at the base of the visual display (as seen in figure 1*a*). Our analysis reveals a slow progressive response increase that plateaus after 300–500 ms (figure 1(iii)). The confounding influence of inhibitory build-up during the control (long trajectory) is likely, if anything, to speed up the rate at which responses to stimuli commencing within the receptive field approach ‘control’ levels. The time course revealed by our normalization thus, if anything, underestimates the underlying response build-up.

**Figure 2.**
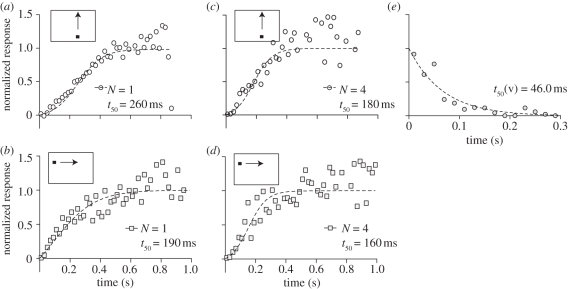
Response half-times. (*a*) Normalized response time course averaged across all start positions (i.e. same data as in figure 1). (*b*) Normalized response onset from the same neuron, to targets drifting horizontally. (*c*) Response to vertical drifts, pooled across four neurons. (*d*) Response to horizontal target drifts, pooled across four neurons. (*e*) The normalized response decay when targets disappeared close to the hotspot (three different receptive field locations, *N* = 1, *n* = 39).

To confirm that the slow onset is not unique to vertical target motion (the average across start positions is shown in figure 2*a*), we use a similar analysis for horizontally drifting targets (figure 2*b*). We recently revealed a strong interaction between CSTMD1 and its contralateral counterpart [15]. This interaction probably affects responses to horizontal target drifts, as these have drifted through the receptive field of the contralateral CSTMD1 before reaching the recorded neuron. Nevertheless, the response to horizontal target trajectories also shows a slow build-up, lasting at least 300–400 ms (figure 2*b*). To confirm that slow response onset is not unique to a particular recording, we pooled data across neurons, and find slow response build-ups to vertical (figure 2*c*) and horizontal target motion (figure 2*d*). We conclude that the slow response facilitation is position and direction invariant.

### (b) Response decay

We previously showed that the velocity tuning of CSTMD1 is well modelled by motion detectors using a relatively brief delay time constant (approx. 40 ms, [12]). Is the slow response build-up simply the result of slow dynamics in the underlying response operating *after* motion detection (i.e. additional low-pass filtering)? To investigate this, we quantified the response *decay* when vertically drifting targets cease motion within the receptive field. After fitting a one-phase decay function to the normalized data (figure 2*e*), we find a half-time of 46 ms, considerably shorter than for onset facilitation. This strong asymmetry in response offset compared with onset argues strongly against a simple low pass filter mechanism.

## 4. Discussion

### (a) Mechanisms underlying facilitation

CSTMD1 is a higher order neuron that does not receive its input directly from elementary STMDs, but indirectly through other STMDs synapsing with its inputs in the lateral mid-brain [15]. Additional synaptic delays on this complex input pathway will increase the initial target detection time and may explain the relatively long absolute latency (figure 1(ii)), but are unlikely to account for the subsequent prolonged response facilitation.

A key finding is that when target motion ceases (figure 2*e*), response offset is dramatically faster than the build-up at response onset. This argues strongly against the most parsimonious explanation for slow onset: that it reflects a simple low-pass filter mechanism operating in higher order neurons that integrate local motion detector outputs, as this would impart sluggishness to both onset and offset, and independent of the contrast of the feature.

An alternative possibility, suggested by the asymmetry in the onset versus offset time course, is that the STMD pathway uses a second order motion detector network (e.g. [18]). Here, a first layer would mediate initial detection of small targets between neighbouring ommatidia (e.g. the elementary small target motion detection scheme [16]). Local target signals would then be processed by a second layer of motion detectors, operating on a larger spatial baseline and with longer neural delays, allowing responses to facilitate to continuous target motion, while preserving small-size selectivity and sensitivity to relatively fast-moving targets. It would also reject noise in local motion detector outputs (since this would not be correlated in space and time), permitting very high amplification. Such a scheme would generate sensitivity to second order motion, even though the stimuli we used here are all first order. Behavioural evidence for second order motion detection, with long response delays (several hundred milliseconds), has been found in *Drosophila* [19].

### (b) Behavioural significance

In dragonflies, behavioural delays to target stimuli are only 25–30 ms [20]. This is very fast compared with the slow response facilitation we have shown here. Nevertheless, CSTMD1 is a higher order neuron that projects to the contralateral lobula [12]. Other dragonfly [14] and hoverfly [21] STMDs have small receptive fields and probably project directly to descending neurons. Barnett *et al*. [21] found that apparent slip in positional information for different target directions was less than 1° at a speed of 50° s^−1^, suggesting effective latency of less than 20 ms, more consistent with behavioural observations [20]. The role of CSTMD1 could potentially be to modulate the gain of small-field-STMDs, or other interneurons, through its contralateral projection. Continuous target trajectories are more likely to represent behaviourally relevant stimuli, ensuring that gain modulation is not initiated by random background scene features. Careful analysis of the absolute response delays of other STMDs and their dependence on parameters such as contrast and size is required in further physiological and behavioural analyses.
